# Differential Inactivation of Fungal Spores in Water and on Seeds by Ozone and Arc Discharge Plasma

**DOI:** 10.1371/journal.pone.0139263

**Published:** 2015-09-25

**Authors:** Min Ho Kang, Anchalee Pengkit, Kihong Choi, Seong Sil Jeon, Hyo Won Choi, Dong Bum Shin, Eun Ha Choi, Han Sup Uhm, Gyungsoon Park

**Affiliations:** 1 Plasma Bioscience Research Center, Kwangwoon University, Seoul, 139–701, Republic of Korea; 2 Department of Crop Life Safety, National Academy of Agricultural Science, Suwon, 441–707, Republic of Korea; 3 Department of Crop Environment, National Institute of Crop Science, Suwon, 441–857, Republic of Korea; Fujian Agriculture and Forestry University, CHINA

## Abstract

Seed sterilization is essential for preventing seed borne fungal diseases. Sterilization tools based on physical technologies have recently received much attention. However, available information is very limited in terms of efficiency, safety, and mode of action. In this study, we have examined antifungal activity of ozone and arc discharge plasma, potential tools for seed sterilization. In our results, ozone and arc discharge plasma have shown differential antifungal effects, depending on the environment associated with fungal spores (freely submerged in water or infected seeds). Ozone inactivates *Fusarium fujikuroi* (fungus causing rice bakanae disease) spores submerged in water more efficiently than arc discharge plasma. However, fungal spores associated with or infecting rice seeds are more effectively deactivated by arc discharge plasma. ROS generated in water by ozone may function as a powerful fungicidal factor. On the other hand, shockwave generated from arc discharge plasma may have greatly contributed to antifungal effects on fungus associated with rice seeds. In support of this notion, addition of ultrasonic wave in ozone generating water has greatly increased the efficiency of seed disinfection.

## Introduction

Fungal infection in plant seeds diminishes the seed vitality and yield, and fungal pathogens can be preserved in seed lots, becoming a causal agent for seed borne diseases [1]. Seed contamination with mycotoxin producing fungi can be a factor threatening human and animal health [2]. Control for seed associated fungal pathogens has been carried out by using fungicides, heat, electrons, natural antifungal products, and biological control agents [3]. Although these methods appear to be effective in controlling seed borne fungal diseases, concomitant drawbacks such as fungicide resistance, pollution, and incomplete efficiency are also followed [3]. Technical improvement and alternative methodologies for efficient disease control are highly needed.

Atmospheric pressure non-thermal plasma and ozone have been applied in sterilization, cancer therapy, skin treatment, and wound healing [4–8]. Plasma is a mixture of ionized gas, producing various ROS and RNS, UV, ions, and the electric field [9], and ozone is a reactive oxygen species with strong oxidizing capacity [5]. Antimicrobial effects of plasma and ozone have been demonstrated in the accumulating number of studies [10–13] and seed disinfection by ozone and plasma has been occasionally reported [6, 14–19]. Sterilization efficiency demonstrated in these studies varies depending on the environments and microbial species. Mode of action in sterilization by ozone and plasma is explained by oxidative stress originated from reactive species, but other factors can also play critical roles depending on circumstances. In order to standardize antimicrobial activity and clarify mechanisms of sterilization by ozone and plasma, systematic analyses are still needed.

In this study, we have examined the antifungal potentiality of plasma and ozone using rice seeds infected with a fungal pathogen causing rice bakanae disease. Rice bakanae disease is one of seed borne diseases, causing great economic loss in rice production [20]. The disease is caused by a fungal pathogen *F*. *fujikuroi* [21]. Rice plants germinated from infected seeds are etiolated and chlorotic, have elongated stems and produce no edible grains [22]. Conventional methodologies for disease control have been focused on seed treatment using hot water, chlorine water, fungicide, and biocontrol [23]. However, disinfection efficiency and emergence of fungicide resistant strains make the traditional treatments less promising. Therefore, a strong requirement for the alternative strategies is on the rise [24]. Our study may provide a crucial information on use of plasma and ozone as potential seed disinfectant(s).

## Materials and Methods

### Fungus and rice seeds used in the study and culture condition

No specific permissions were required for using infected rice seeds because seeds were provided by the National Institute of Crop Science of Rural Development and Administration (RDA) in the Republic of Korea. During flowering time, spore suspension of *F*. *fujikuroi* (10^6^ /ml) was sprayed onto rice plants (*Oryza sativa* L. cv. *Hopyeong*) cultivated in an authorized research greenhouse at the National Institute of Crop Science. After harvested, seeds confirmed to be infected with *F*. *fujikuroi* were received and used in experiments. Seeds were kept at 4°C in the dry condition until used for experiments. A fungal strain causing bakanae disease, *F*. *fujikuroi* CF284 was generously provided by RDA in the Republic of Korea. Fungal spores and mycelia were stored in 20% glycerol solution at -80°C. The fungal strain was propagated and grown on Potato Dextrose Agar (PDA) plate at 25°C in the dark.

### Ozone and plasma generating systems

**Fig 1:**
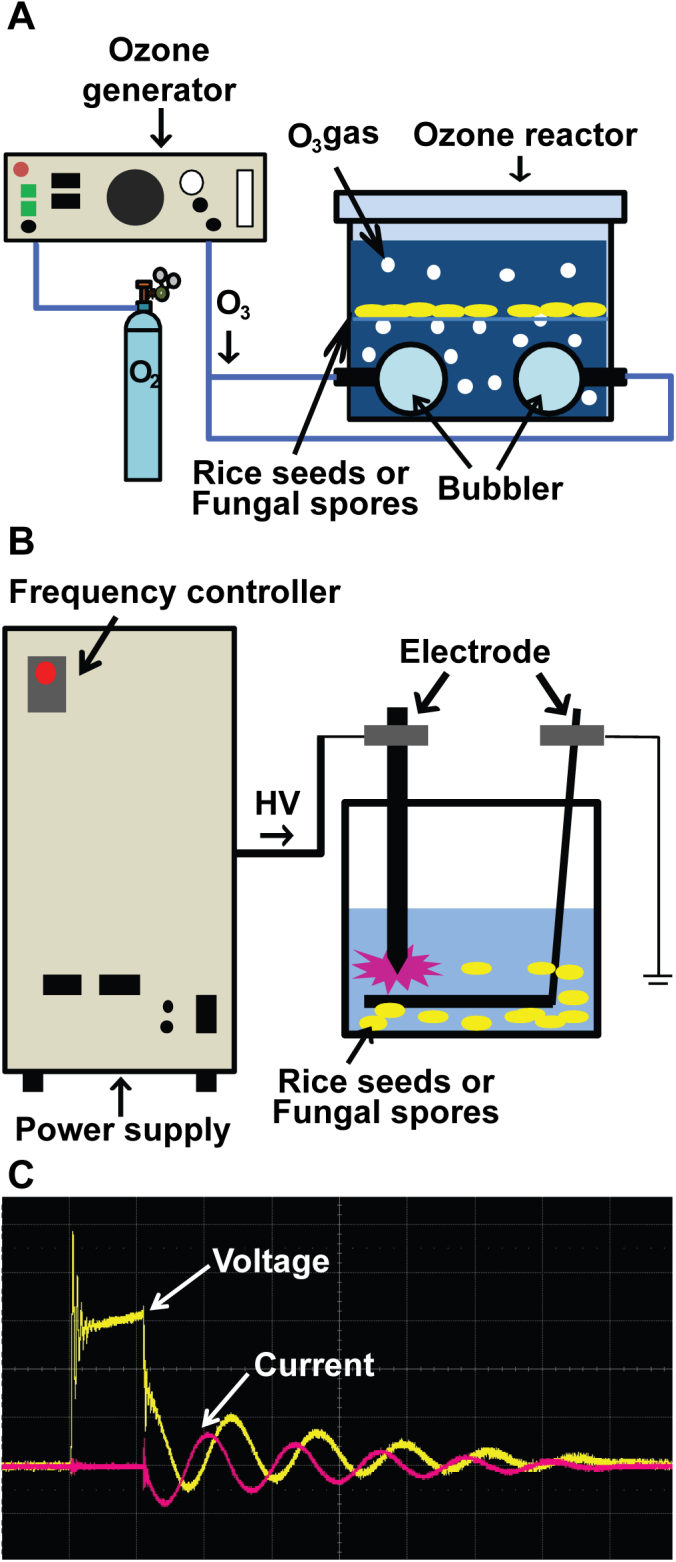
(color online) Schematic view of the devices and treatment setting. A. Set-up for ozone treatment. Ozone generator was connected to an acryl container filled with fungal spore suspension or rice seeds. B. Set-up for treatment with arc discharge plasma. A high voltage DC power supply was connected to two electrodes submerged in spore or rice seeds suspension in the container. C. Voltage and current profile during arc discharge at 10 kV and 12 Hz.

Device setting for ozone generation was carried out as previously described [11]. As shown in Fig 1A, ozone generator was connected to an acryl square container (115x85x105 mm) by tygon tube, and generated ozone was supplied into the container through tube line. To produce ozone, oxygen gas was provided to the generator with 1 lpm (liter per minute) flow rate, and 217 V and 0.97 A electricity was applied. The level of ozone was measured by using an analyzer OZM-7000 (Ozonetech, Daegu, Republic of Korea). The maximum level of ozone gas was passed through tube line and provided into water in the acryl container (where fungal spores or rice seeds were placed) using bubbler (Fig 1A).

Arc discharge plasma system was composed of two tungsten electrodes and a DC high-voltage power supply (Fig 1B). One of electrodes had needle shape that was connected to the power supply (Ksc, Seoul, Republic of Korea) and the other board electrode was functioned as ground (Fig 1B). Two electrodes were submerged into water in an acryl square container (60 x 85 x 50 mm) and distance between electrodes was 1 mm (Fig 1B). Plasma was generated in water by arcing between two electrodes discharged at 10~15kV. Voltage and current during arc discharge were monitored using oscilloscope (Fig 1C). Discharge frequency was controlled by frequency controller located in DC high-voltage power supply.

### Treatment with ozone, arc discharge plasma and ultrasonic wave

To treat fungal spores with ozone or arc discharge plasma, spores were produced and harvested. Mycelial pieces of *F*. *fujikuroi* grown on PDA plate for 2 weeks were inoculated in 1 L of VM (Vogel’s Minimal) liquid media and the flask containing inoculated media was incubated at 25°C with shaking for 5 days. Then, spores were collected by filtering liquid culture through 3 layers of miracloth (EMD Millipore Co., Billerica, MA, USA) and centrifuged at 4000 rpm for 5 min. After washing fungal spores with sterile water, collected spores were resuspended in new sterile water (10^7^ / ml, total 200 ml). Spore suspension was placed in acryl square container connected to ozone generator or arc discharge plasma system and then generated ozone or plasma was applied to spore suspension for indicated time. Spores suspended in water with no treatment were used as control. After treated with ozone or plasma, whole spore suspension was transferred to 50 ml tubes and tubes were centrifuged at 4000 rpm for 5 min. Spores pelleted in several tubes were combined in a tube and resuspended in sterile water. After proper dilution, spore suspension was spread on PDA to assess germination.

For treatment of rice seeds with ozone or arc discharge plasma, 75 infected rice seeds provided by the National Institute of Crop Science were submerged in water placed in an acryl square container and then exposed to ozone or arc plasma for indicated time. For control, same number of rice seeds incubated in water without treatment was used.

Ultrasonic wave was produced in ozone generating water by ultrasonicator in order to examine the effect of shockwave on seed disinfection. Rice seeds harvested from the disease outbreak place were put in the container with ozone generating water and the container was placed in the ultrasonicator. Ultrasonic wave was generated under 40 kHz frequency and 350 W power.

### Assessment of fungal inactivation

After exposure to ozone or arc discharge plasma, fungal spores were pelleted down by centrifugation at 4000 rpm for 5 min and then resuspended in new water as described in the earlier section. Spore suspension was diluted and spread onto PDA plate. After 2–3 days, number of germinated spores (appeared as colonies; CFU) was counted.

Rice seeds after treated with ozone, arc discharge plasma or ozone + ultrasonic wave were placed on *F*. *fujikuroi* selective minimal media (containing 2.5 mM PCNB; Pentachloronitrobenzene and 3 mM hydrogen peroxide). Media plates (25 seeds per plate; 90 mm petri dish) were incubated at room temperature for 5–6 days and then fungal growth around seeds was monitored. Seeds with no fungal growth were counted as disinfected ones.

### Analysis using electron microscope

Surface and internal structures of fungal spores were analyzed using scanning and transmission electron microscopes (SEM and TEM). After treated with ozone or arc discharge plasma, fungal spores were collected by centrifugation at 5000 rpm for 5 min, washed with 1X PBS, twice, and then fixed in Karnovsky’s fixative overnight. Fixed fungal spores were further processed as described previously [25]. Prepared samples were examined under SEM (JEOL, Tokyo, Japan) and TEM (JEOL, Tokyo, Japan).

### Measurement of pH and temperature

After treatment with ozone and plasma, pH of solutions was measured using a portable pH meter (Eutech Instruments, Singapore). Temperature of water was measured by placing alcohol thermometer into water immediately after exposure to ozone or arc plasma for indicated time. All measurements were carried out in triplicates.

### Assay for ROS and RNS concentration in the treated water

ROS (OH radical, singlet oxygen) and RNS (nitric oxide, peroxynitrite) in water were measured by using H2DCF (2',7'-dichlorodihydrofluorescein, major species; OH radical and peroxynitrite, Life Technologies, Carlsbad, California, USA), DAF-FM (4-amino-5-methylamino-2′,7′-difluorofluorescein, major species; nitric oxide, Life technologies, Carlsbad, California, USA), and singlet oxygen green reagent (singlet oxygen; Life technologies, Carlsbad, California, USA). H2DCF was obtained by incubating H2DCFDA (2’,7’-dichlorodihydrofluorescein diacetate) in 0.05 M NaOH for 30 min at 37°C in the dark [26]. Ozone dissolved in water was measured using an aqueous ozone analyzer (OZM-7000; Okitrotec Co. Ltd., Tokyo, Japan). Same volume of water as used in the treatment of fungal spores or rice seeds was exposed to ozone or arc discharge plasma under indicated conditions, and then 1 ml of treated water was transferred to a microcentrifuge tube. Chemical indicators were immediately added into the tube (final concentration 10 μM) and then water reacted with chemical indicators was transferred to a 96-well fluorescent plate (100 μl per well). The plate was read at 495/515 (ex./em. for H2DCF and DAF-FM) and 504/525 nm (ex./em. for singlet oxygen). For ozone measurement, 1 ml of treated water was transferred to ozone analyzer and concentration was read.

### Statistical analysis

Statistical analysis of the data was performed using the Student’s *t* test to determine significance between data points. Significant differences were established at *p*<0.05 or *p*<0.01 (*denotes p<0.05 and ** denotes p<0.01).

## Results

### Ozone inactivates fungal spores more efficiently

**Fig 2:**
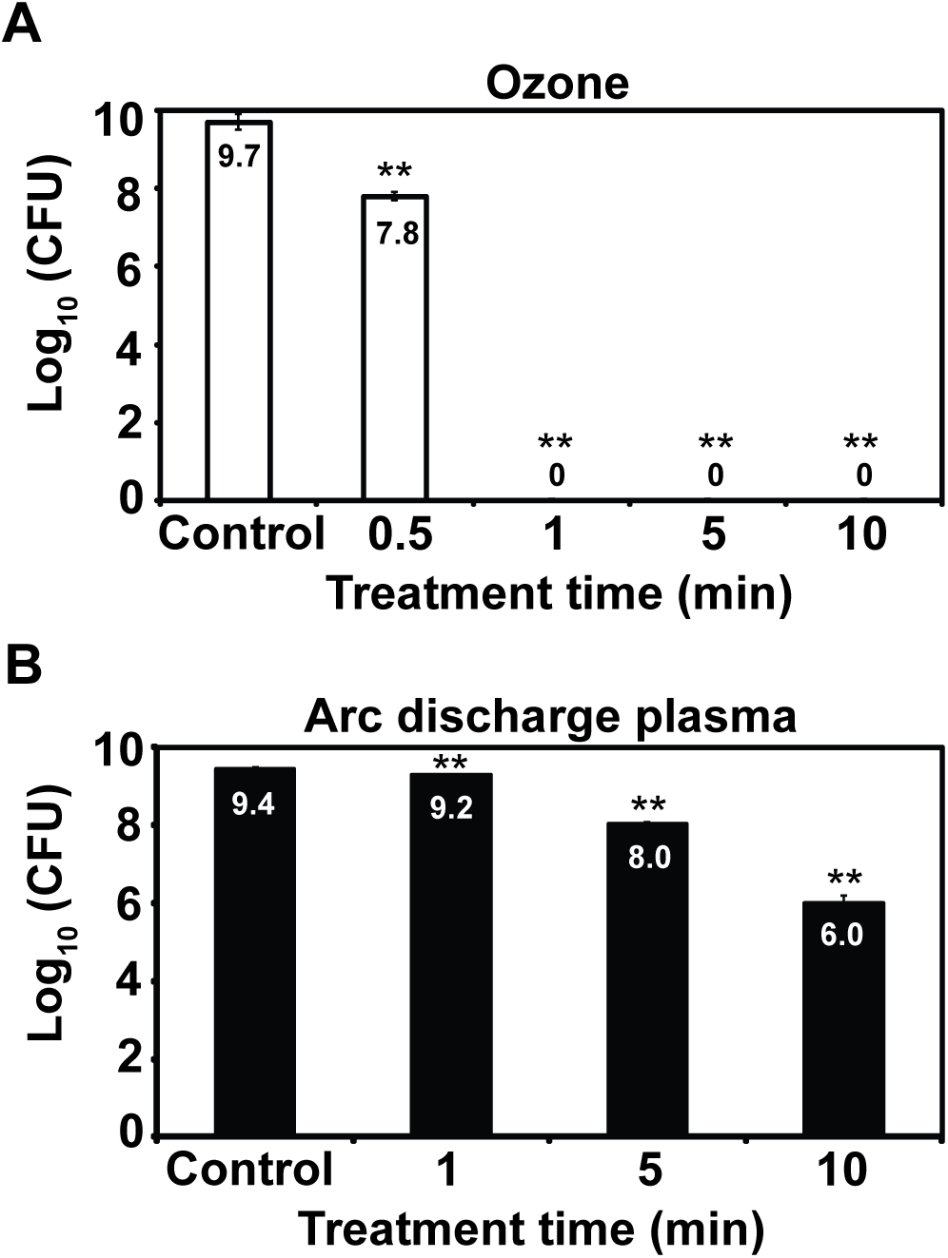
Germination of fungal spores after ozone or plasma treatment. Germination of *F*. *fujikuroi* spores after treatment with ozone (A) or arc discharge plasma (B). Germinated fungal spores were appeared as colonies on PDA plate and the number of colonies (CFU) was counted and converted to the log scale. Each value was an average of 3 experimental replicates and indicated on the bar. Student t-test was performed between control and each treatment; ** p < 0.01.

When ozone was applied to spores of *F*. *fujikuroi* in water (total number 2 x 10^9^), number of germinated spores (indicated as log value of colony forming unit number) after treatment was dramatically decreased in treatment time dependent manner (Fig 2A). Most of spores became inactive after a 1 min treatment (Fig 2A). When fungal spores (total number 2 x 10^9^) were treated with plasma generated by arc discharge (12 KV, 3 Hz) in water, the number of germinated spores was also decreased in treatment time dependent manner but about 4 log reduction in the number of active spores was observed after a 10 min treatment (Fig 2B). Overall, spore inactivation was much greater in the treatment with ozone than arc discharge plasma upon same treatment time (Fig 2).

**Fig 3:**
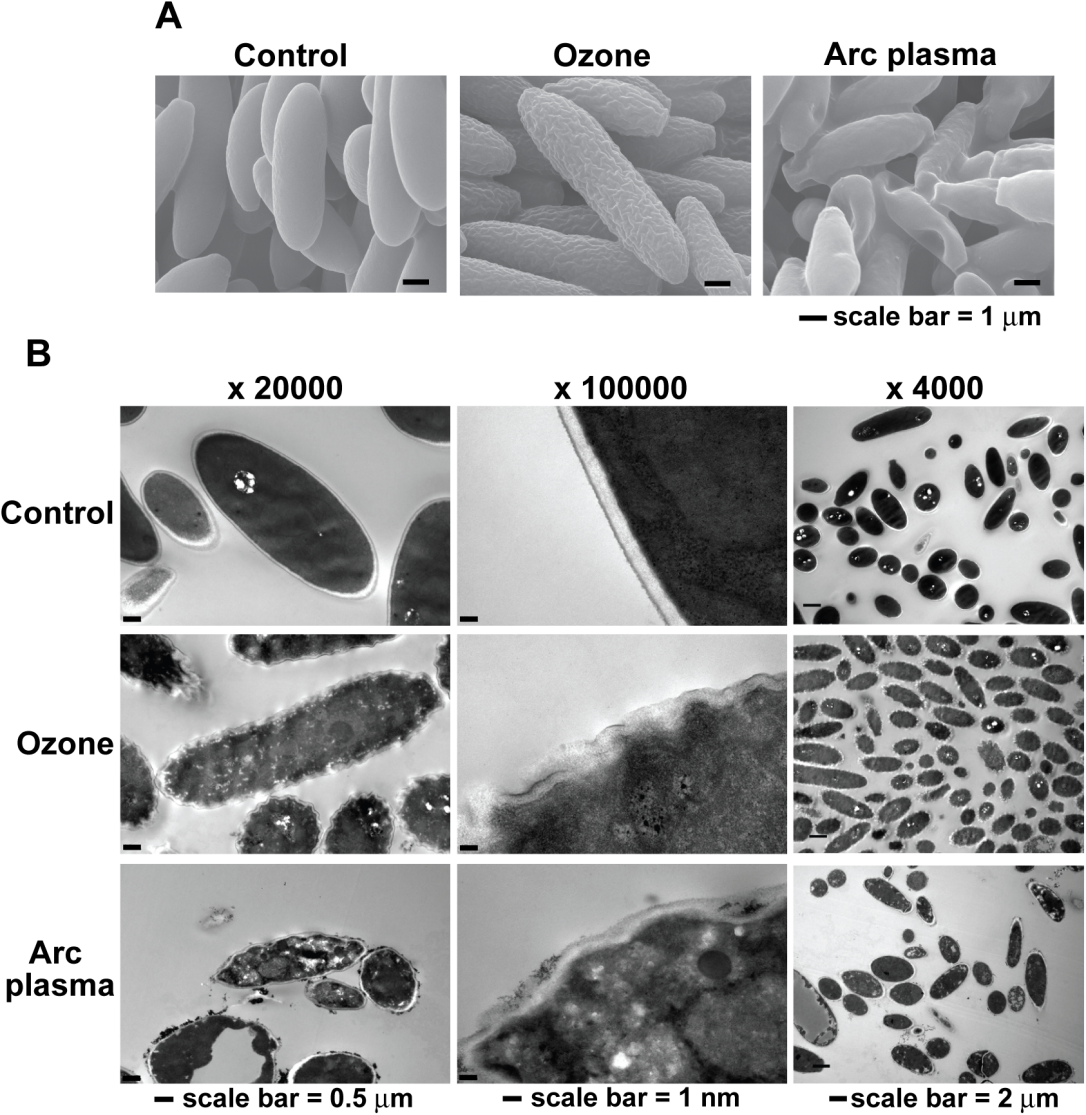
Structure of fungal spores after ozone or plasma treatment. Morphology and internal structure of spores analyzed by using SEM (A) and TEM (B) after ozone and arc discharge plasma treatment. Fungal spores in water (10^7^ / ml, total 200 ml) were treated with ozone for 30 sec or arc plasma for 10 min as described in materials and methods. After treatment, spores were collected by centrifugation and processed for electron microscopy.

SEM and TEM analysis demonstrate that ozone and arc discharge plasma can cause different structural modifications on fungal spores. Spores treated with ozone had wrinkled surfaces with no dramatic change in shape whereas those treated with arc discharge plasma were crushed and shrinked compared to control (Fig 3A). In TEM analysis, cytoplasm of ozone treated spores was slightly less electro-dense than that of control, and organelles were not severely damaged (Fig 3B). However, many spores treated with arc discharge plasma exhibited much less electro-dense cytoplasm with empty spaces and more destroyed organelles (Fig 3B). More severe degeneration was observed in surface structure of spores treated with ozone but arc discharge plasma affected more internal structure of spores (Fig 3B). Both ozone and arc plasma treatment caused damage on spore surface (probably cell membrane and wall). In control spores (no treatment), cell surface was appeared as clear black lines distinguished from intracellular area (Fig 3B middle panel). These lines were destroyed after ozone and arc plasma treatment and more destruction was observed in spores treated with ozone (Fig 3B middle panel).

### Arc discharge plasma disinfects rice seeds more efficiently

**Fig 4:**
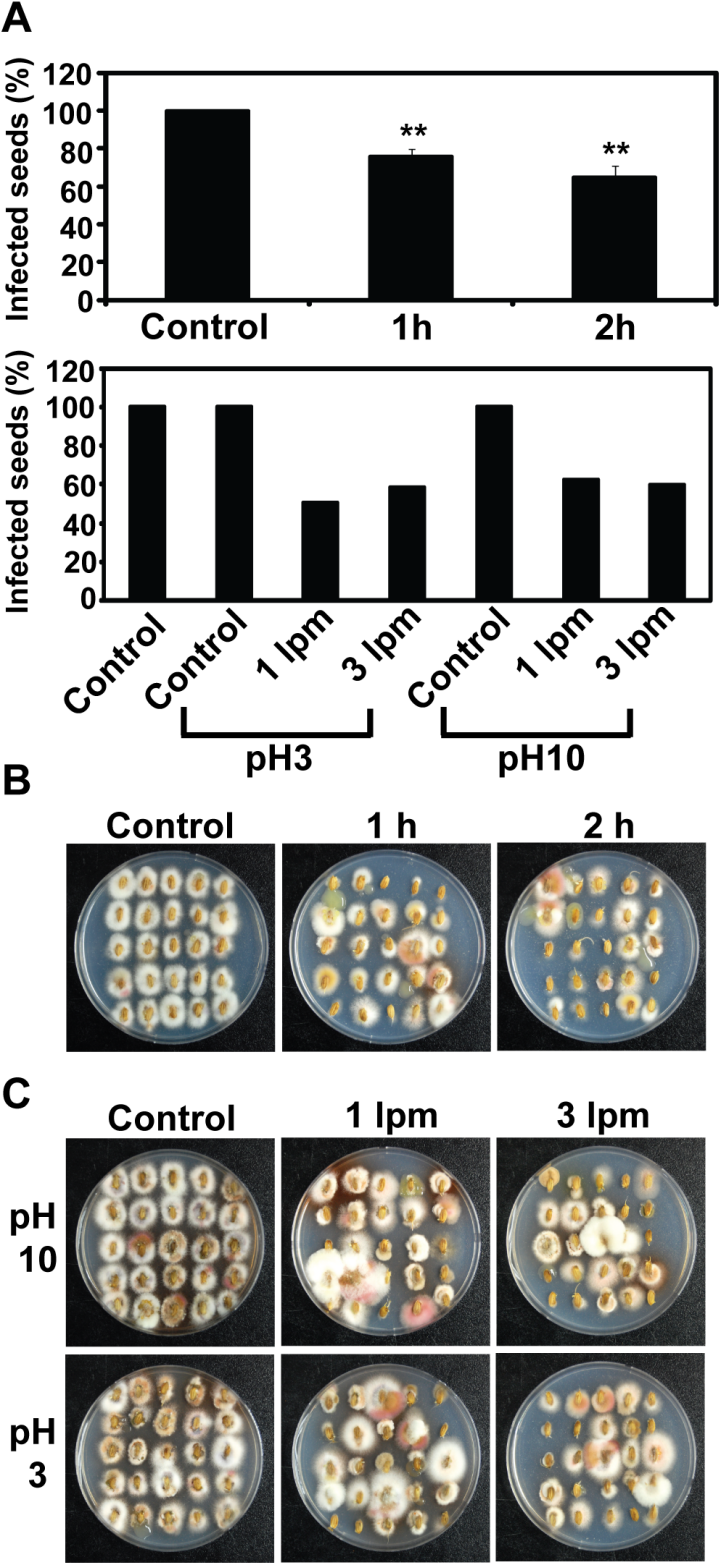
(color online) Disinfection of rice seeds by ozone treatment. A. Percentage of infected seeds after treated by ozone in water for 1 and 2 hrs (upper graph) or in water of different pH (3 and 10) and with different ozone injection rate (1 and 3 lpm) for 2 hrs (bottom graph). In upper graph, each value was an average of 3 experimental replicates. Student t-test was performed between control and each treatment; ** p < 0.01. B. Fungal growth from rice seeds after treated with ozone in water for 1 and 2 hrs. C. Fungal growth from rice seeds after treated with ozone in water of different pH (3 and 10) and ozone injection rate (1 and 3 lpm) for 2 hrs. All plates were incubated at 25°C for 6 days and then imaged.

Since more fungal spores are inactivated by ozone than arc discharge plasma in water, it is expected that rice seeds may be more efficiently disinfected by ozone. We treated infected rice seeds received from the National Institute of Crop Science with ozone or arc discharge plasma. Total 75 seeds were treated in water by ozone or arc discharge plasma as described earlier. Disinfection of seeds was assessed by checking fungal growth from the treated seeds after incubated on *F*. *fujikuroi* selective minimal media for 5–6 days. About 50% of seeds was still infected with fungus after treatment with ozone in water (1 lpm injection rate) for 1 and 2 h (Fig 4A upper graph) and growth of fungal mycelia was still observed around many treated seeds (Fig 4B). Disinfection rate was not significantly affected by pH of water or ozone injection speed. About 40–50% disinfection rate was obtained from 2h ozone treatments in water of different pH (3 and 10) or with different injection rate (1 and 3 lpm) (Fig 4A bottom graph and 4C).

**Fig 5:**
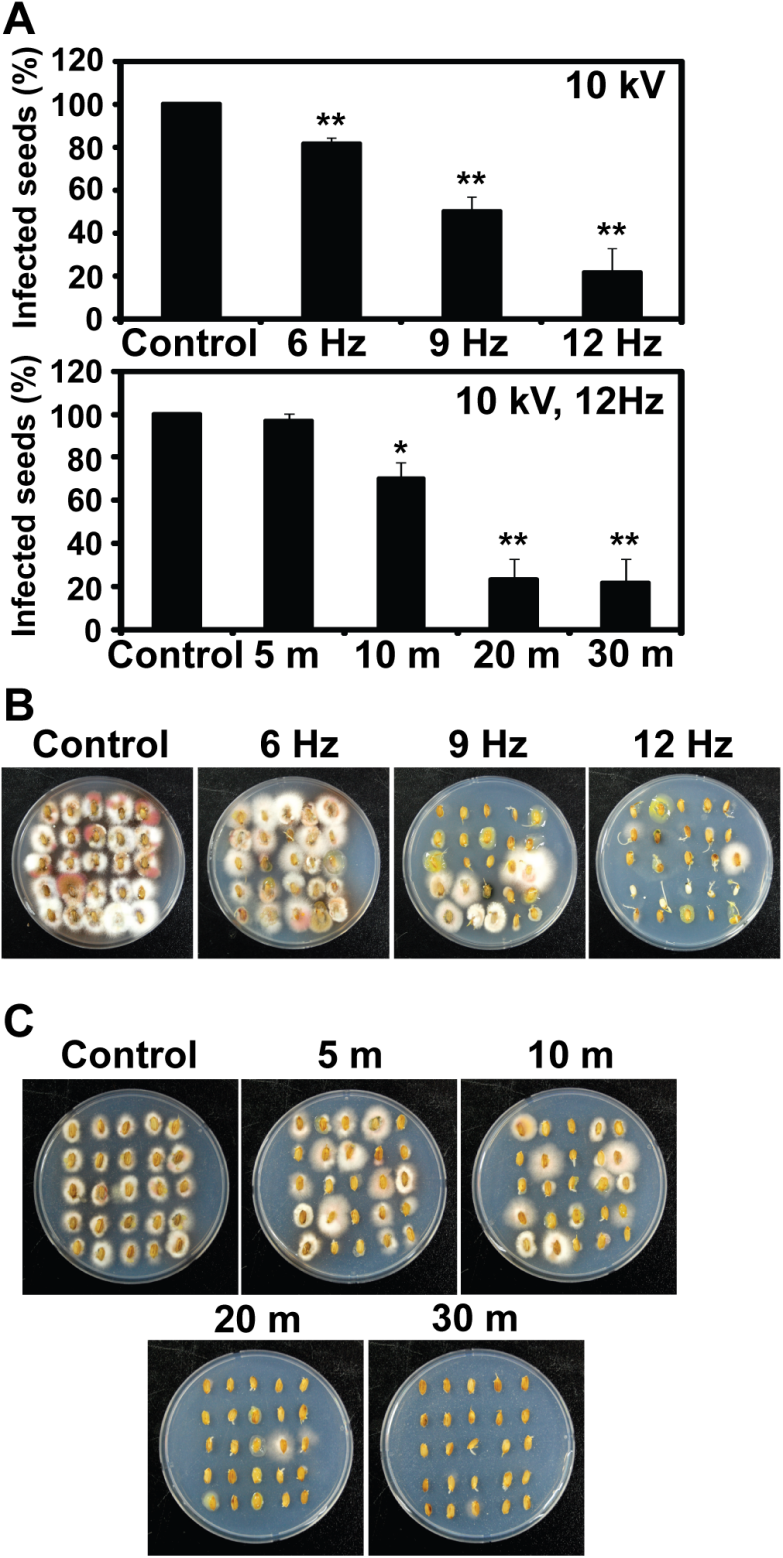
(color online) Disinfection of rice seeds by arc discharge plasma treatment. A. Percentage of infected seeds after treated in water by arc plasma discharged at 10 kV and different frequencies (6, 9, 12 Hz) for 30 min (upper graph) or discharged at 10 kV and 12 Hz for 5, 10, 20, and 30 min (bottom graph). Each value was an average of 3 experimental replicates. Student t-test was performed between control and each treatment; * p< 0.05, ** p < 0.01. B. Fungal growth from rice seeds after treated with arc plasma discharged at 10 kV and different frequencies (6, 9, 12 Hz) for 30 min. C. Fungal growth from rice seeds after treated with arc plasma discharged at 10 kV and 12 Hz for 5, 10, 20, and 30 min. All plates were incubated at 25°C for 6 days and then imaged.

Interestingly, arc discharge plasma exhibited much greater efficiency in disinfecting rice seeds than ozone. When seeds were treated with arc plasma (discharged at 10 kV), disinfection rate was increased proportionally to the elevation of discharge frequency (Fig 5A upper graph). About 80% of rice seeds was disinfected by treatment with arc plasma discharged at 12 Hz for 30 min (Fig 5A upper graph). Majority of rice seeds treated with 12 Hz discharge plasma exhibited fungus-free on selective media (Fig 5B). In order to get over 80% sterilization efficiency, rice seeds had to be exposed for at least 20 min (Fig 5A bottom graph). Dramatic reduction in fungal growth from seeds was observed on selective media after treatment with 12 Hz discharge for 20 and 30 min (Fig 5C).

### Different factors may be crucial for inactivation of planktonic or seed associated fungal spores

**Fig 6:**
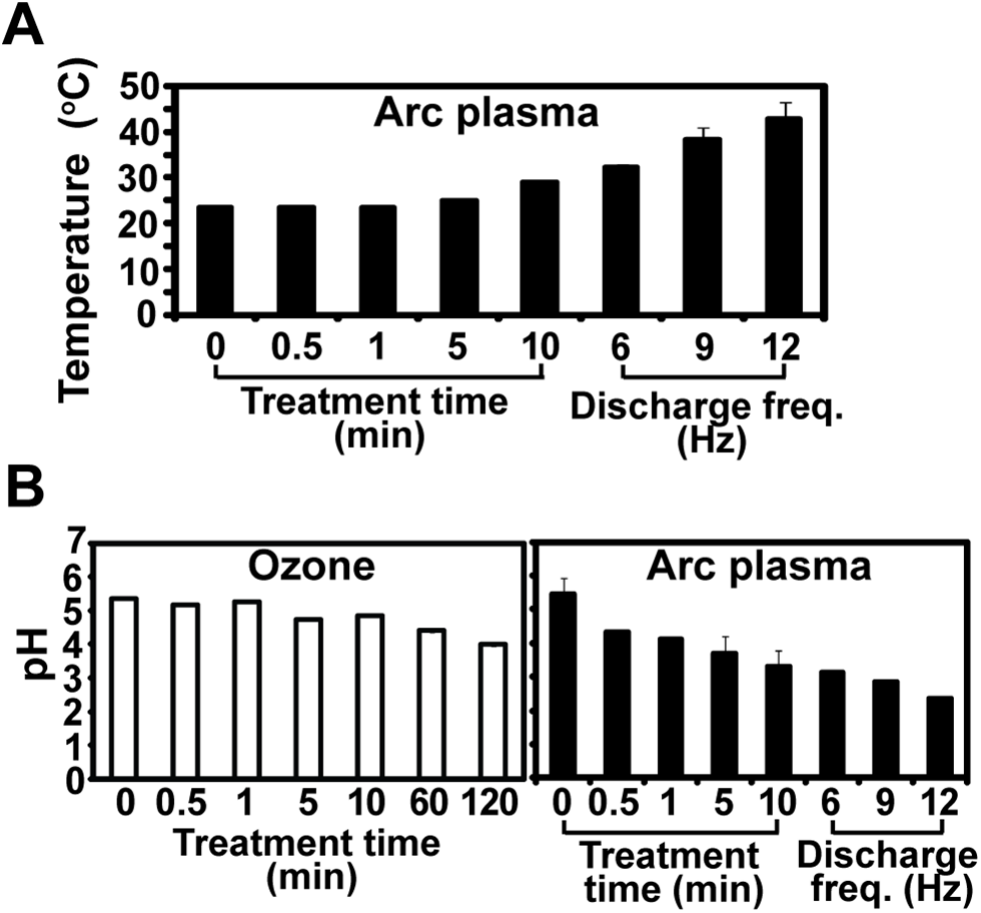
Measurement of temperature and pH in water after ozone or arc discharge plasma treatment. A. Temperature of water after treatment with arc discharge plasma. Arc plasma was discharged at 10 kV and 12 Hz for 0.5, 1, 5, and 10 min or at 10 kV and different frequencies (6, 9, 12 Hz) for 30 min. B. pH of water after treated with ozone or arc discharge plasma for indicated time. Ozone injection rate was 1 lpm. Arc plasma was discharged at 10 kV and 12 Hz for 0.5, 1, 5, and 10 min or at 10 kV and different frequencies (6, 9, 12 Hz) for 30 min.

As shown in our results, arc discharge plasma was much more effective in disinfecting rice seeds although not as efficient as ozone in inactivating fungal spores. To identify factor(s) determining antifungal effects of ozone and arc plasma, physical and chemical properties of water such as temperature, pH and the level of reactive species were analyzed after treatment with ozone or arc plasma. Temperature of water was maintained at around 25°C during ozone treatment (data not shown) but increased to around 45°C after treatment with 12 Hz discharged arc plasma (Fig 6A). This range of temperature may not critically affect the viability of fungal spores because it is lower than 60°C used in seed treatment for disease control by heat [21]. However, accumulated effect of increased temperature on both fungus and seed vitality should be also considered. pH of water was decreased to around 2.5 (from around 6 for control) after treatment with arc discharge plasma (at 12 Hz for 30 min) and to around 4 after ozone treatment for 2 hrs (Fig 6B). Acidification of water by plasma has been often observed in studies and can contribute to fungal inactivation although it is not considered as a main microbicidal factor [27–28].

**Fig 7:**
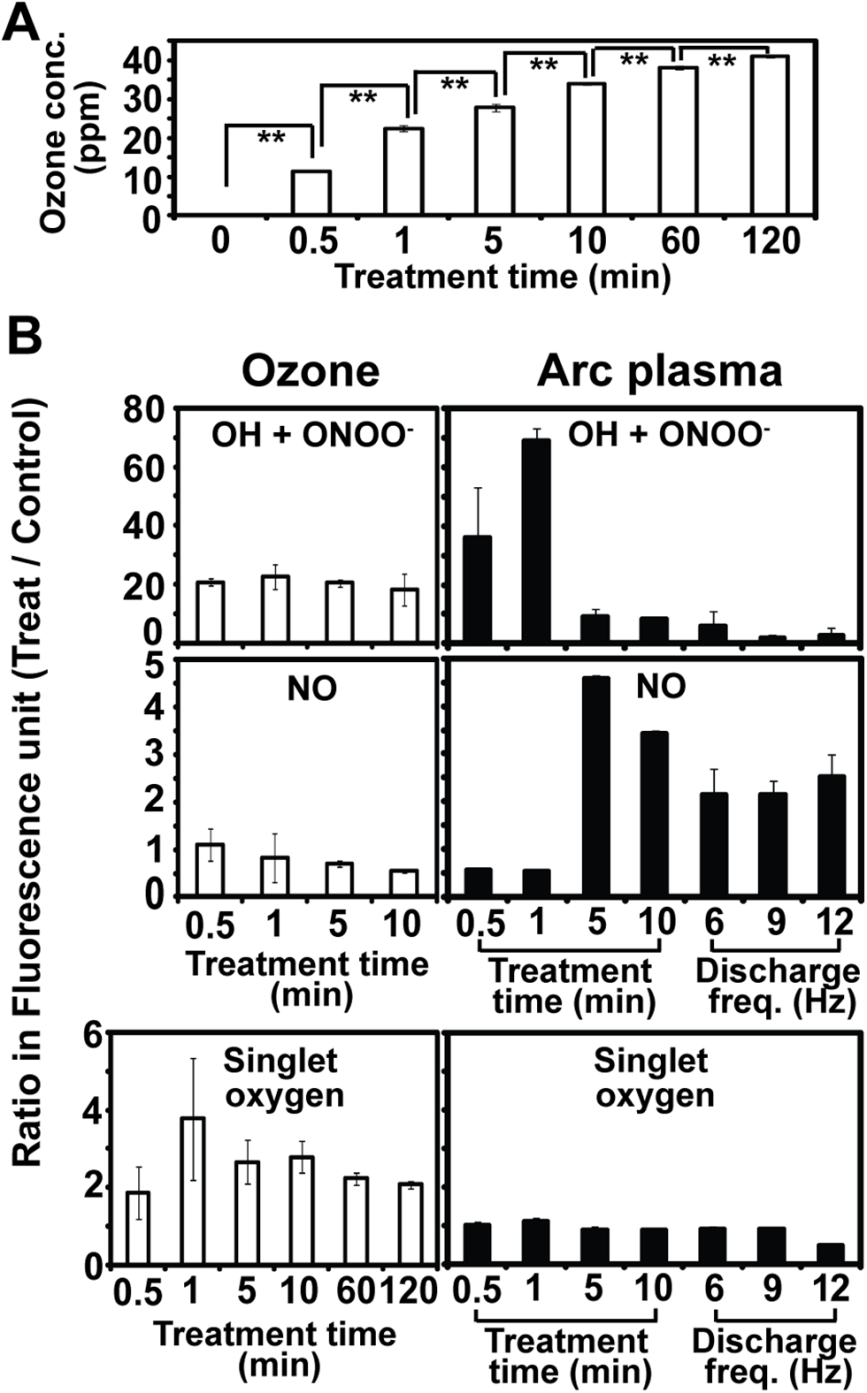
Level of reactive species in water after ozone and arc discharge plasma. A. Concentration of ozone (ppm) present in water after ozone injection (1 lpm) for indicated time. All measurements were performed in 3 replicates. Student t-test was performed between each treatment time; ** p < 0.01. B. Relative level of ROS (OH radical, peroxynitrite, singlet oxygen) and RNS (nitric oxide) in water after ozone or arc plasma treatment compared to control (no treatment). All values on y-axis are the ratio between treatment and control (ratio = OD_wavelength_ of ozone or plasma treatment / OD_wavelength_ of control). All measurements were performed in 3 replicates.

In the treatment with ozone, level of ozone dissolved in water was significantly increased over time and reached to about 40 ppm after 1 hr (Fig 7A). ROS level measured by H2DCF (mainly OH radical and peroxynitrite ONOO^-^) was dramatically increased (20 times) after 30 sec treatment with ozone and then the level was maintained during treatment (Fig 7B left panel). Level of singlet oxygen was at least 2 times higher in ozone treatment compared to control (Fig 7B left panel). However, there was no increase in nitric oxide concentration in water treated with ozone (Fig 7B left panel). It seems that OH radical, peroxynitrite, and singlet oxygen were quickly saturated in 200 ml of water after ozone treatment.

Generally, water treated with arc discharge plasma exhibited no dramatic change in ROS (OH radical, peroxynitrite, singlet oxygen) level during treatment (Fig 7B right panel) although relative amount of OH radical and peroxynitrite was increased in the treatment for 30 sec and 1 min (Fig 7B right panel). No increase in ROS level was observed in the discharge condition that produced the highest disinfection efficiency (12 Hz discharge). Relative level of nitric oxide was increased after treatment for at least 5 min (Fig 7B right panel). After exposure to arc plasma discharged at 10 kV for 30 min, nitric oxide level in water became 2 times higher, and similar level was observed among treatments with different discharge frequencies (6, 9, 12 Hz) (Fig 7B right panel).

### Addition of shockwave increases efficiency of seed disinfection by ozone

**Fig 8:**
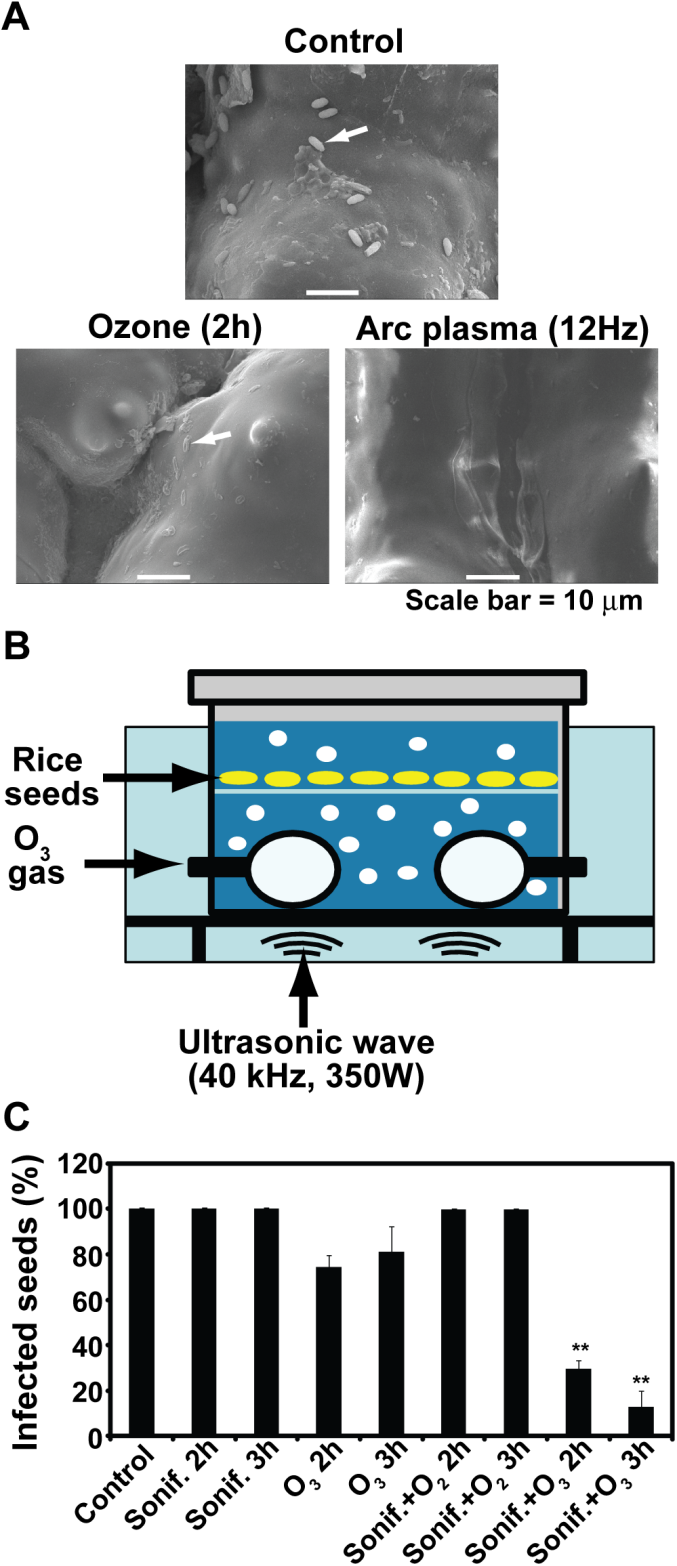
SEM analysis of seed surface after ozone or arc discharge plasma. A. Surface of rice seeds after treated with ozone (2 h) or arc discharge plasma (10 kV, 12 Hz for 30 min). Rice seeds with no treatment were used as control. Arrows indicate fungal spores. B. Set up for treatment with both ultrasonic wave and ozone in water. Ultrasonic wave was generated at 350 W power and 40 kHz frequency. C. Percentage of infected seeds after treated in water by both ultrasonic wave (indicated as Sonif.) and ozone (O_3_) for 2 or 3 hrs. Each value was an average of 3 experimental replicates. Student t-test was performed between control and each treatment; ** p < 0.01.

Interestingly, we observed that fungal spores were hardly found on the surface of rice seeds after treated with arc discharge plasma while traces of more spores were observed on the surface of ozone treated rice seeds (Fig 8A). In the ozone treatment, we could not see many intact fungal spores on seed surface as shown in control, but structures like traces of spores were more often observed (Fig 8A). It might be possible that spores left on the surface of ozone treated seeds were come off by chemical treatment during SEM sample preparation. Nevertheless, the result indicates that arc plasma may facilitate detachment of fungal spores from seed surface.

It is known that arc discharge plasma can produce shockwave during discharge in water [29]. We hypothesize that shockwave generated by arc discharge plasma may have played an important role in detachment of fungal spores from seeds. Detached spores are then probably inactivated by reactive species generated from arc plasma. Both mechanical wave and reactive species may be essential for efficient inactivation of fungal spores associated with hydrophobic seed surface. To further examine this hypothesis, we used ultrasonic wave as a source of shockwave and ozone as a strong inactivator for fungal spores. (Fig 8B). Ultrasonic wave can produce similar effects as shockwave does. When seeds were treated in both ozone and ultrasonic wave generating water, the number of disinfected seeds was significantly elevated (Fig 8C). About 90% disinfection rate was obtained after treatment with both ultrasonic wave and ozone in water for 3 hrs (Fig 8C). However, treatment with only ozone, oxygen and ultrasonic wave, or only ultrasonic wave resulted in less than 50% seed disinfection (Fig 8C). This result indicates that additional treatment with ultrasonic wave in ozone generating water can improve the sterilization efficiency compared to treatment with ozone only or ultrasonic wave only.

## Discussion

Our data demonstrate that treatment with ozone or arc discharge plasma can result in different level of fungal inactivation depending on where spores are associated. Planktonic fungal spores in water can be easily inactivated by ozone but arc discharge plasma is more effective in eradicating fungal spores associated with rice seeds. Antimicrobial effects of plasma and ozone have often shown a broad spectrum of efficiency depending on microbial species and environment [30–33]. Search for the efficient plasma sources and combination with other technologies may be necessary on a case by case basis, and our results may provide an useful information on developing efficient plasma sources and methodologies.

As demonstrated in our results, factors that are critical for fungal inactivation may be different between rice infected and free living spores in water. Studies have suggested that reactive species, UV, and electrical field from plasma can play major roles in sterilization [34] [9, 35]. In our results, ozone is a strong killer of fungal spores freely submerged in water. In addition, ozone treated water has much higher amount of OH radical and singlet oxygen than arc plasma treated water. Although arc discharge plasma generates more nitric oxide in water than ozone, it inactivates fungal spores with much less efficiency than ozone. This indicates that nitric oxide produced by arc plasma treatment may be less critical in fungal spore inactivation than ROS produced by ozone such as ozone, OH radical, and singlet oxygen. Therefore, oxidative stress from ozone generated ROS to fungal spores may be much greater than that from plasma generated nitric oxide.

Our data also indicate that ozone is not enough for disinfection of seeds with hydrophobic surface like rice seeds although it is a very strong inactivator of fungal spores. This is probably because surface of rice seed is hydrophobic and bumpy, and so ozone dissolved water cannot reach fungal spores efficiently. On the other hand, shockwave produced from arc discharge plasma may provide mechanical power to detach spores or carry reactive species to every part of seed surface. Thus, reactive species generated by arc discharge plasma can contact rice seeds more efficiently. After come off from seed surface, fungal spores may encounter reactive species (mostly RNS) and acidic pH, and are eventually inactivated. Our SEM analysis shows almost no spores left on the surface of rice seeds after treated with arc discharge plasma. In addition, shockwave may transfer a mechanical shock to spores causing deformation in shape [29]. Many spores were crushed in shape after treated with arc discharge plasma as shown in our SEM analysis.

Since ozone and shockwave are likely to be major antifungal factors in our experiments, combination of these two may produce the synergistic effect. Addition of ultrasonic wave in ozone provided water was more efficient in seed disinfection than ozone only or arc discharge plasma as shown in our result. This result implicates that search for an efficient combination of antimicrobial tools to generate synergistic effect may be required for the control of seed borne diseases. Since *F*. *fujikuroi* spores can germinate and penetrate inside rice seeds, disinfection of seeds internally is an another issue to be solved in disease control. In a severely infected rice seed, about half of fungal spores are found in internal part of seed [21]. To date, there is no efficient tool for inactivating fungus inside seed. In our results, no fungal growth from about 80% of arc plasma treated seeds was observed even after 6 day incubation on selective media (Fig 5). Similar results were observed in seeds treated with both ultrasonic wave and ozone. Incubation time of 6 days may not be enough to determine fungal inactivation inside seeds but our data suggest a possibility that treatment with arc discharge plasma or with both ultrasonic wave and ozone can potentially eradicate fungus within seeds. These issues are currently under investigation by authors.

About effect of ozone, arc plasma and ultrasonic wave on seed vitality, quantitative analysis on seed germination and growth after treatment is ongoing. Our preliminary observations demonstrate that seeds can still germinate even on media after arc plasma treatment (see Fig 5), and 100% of seeds (100 seeds tested) are germinated after treatment with both ultrasonic wave and ozone like control.

## Conclusions

We demonstrated differential inactivation of *F*. *fujikuroi* spores submerged in water or associated with rice seeds by ozone, arc discharge plasma or ultrasonic wave. Spores in water were more efficiently deactivated by ozone whereas arc discharge plasma disinfected rice seeds more effectively. ROS produced in water by ozone may be effective in inactivating fungal spores, whereas shockwave generated by arc discharge plasma may have played an important role in eradicating *F*. *fujikuroi* on rice seeds. Addition of ultrasonic wave in ozone treatment disinfected rice seeds more efficiently than ozone or arc discharge plasma treatment. Combination treatment using ultrasonic wave and ozone should be further investigated for developing efficient and eco-friendly control strategies of rice bakanae disease.
